# Case Report: Ectopic Cushing’s syndrome caused by a pancreatic neuroendocrine tumor managed through dual steroidogenesis inhibition and endoscopic ultrasound-guided ethanol ablation

**DOI:** 10.3389/fendo.2025.1700273

**Published:** 2025-12-18

**Authors:** Łukasz Krzystek, Karol P. Sagan, Elżbieta Andrysiak-Mamos, Szymon Retfiński, Karolina Buć, Wiktoria Marczak, Anna Brzeska, Martyna Patalong-Wójcik, Ewa Żochowska, Krzysztof Dąbkowski, Anhelli Syrenicz

**Affiliations:** 1 Pomeranian Medical University, Szczecin, Poland; 2 Department of Endocrinology, Metabolic and Internal Diseases, Pomeranian Medical University, Szczecin, Poland; 3 Department of Gastroenterology, Pomeranian Medical University, Szczecin, Poland

**Keywords:** ectopic Cushing’s syndrome, pancreatic neuroendocrine tumor, ACTH, hypercortisolism, osilodrostat, etomidate, ethanol ablation

## Abstract

Ectopic adrenocorticotropic hormone (ACTH)-dependent Cushing’s syndrome (EAS) is a rare complication of neuroendocrine tumors (NETs). Severe hypercortisolism (SH) requires urgent medical intervention due to its life-threatening consequences. We report a 74-year-old female patient with an ACTH-secreting pancreatic NET (pNET) who presented with rapidly progressive cognitive decline, muscle weakness, severe hypokalemia, and hyperglycemia. Laboratory evaluation confirmed ACTH-dependent Cushing’s syndrome with loss of diurnal cortisol rhythm and panhypopituitarism. Surgical treatment was contraindicated because of significant comorbidities. The initial management included intravenous etomidate infusion. Subsequently, osilodrostat was introduced as long-term oral therapy. Marked clinical and hormonal improvements were observed, including the normalization of potassium and cortisol levels, resolution of neuropsychiatric symptoms, and restoration of mobility. After 19 months of osilodrostat therapy, endoscopic ultrasound-guided ethanol ablation of the pancreatic lesion was performed, and medical therapy was discontinued. This case demonstrates the effectiveness of dual steroidogenesis blockade with etomidate and osilodrostat in both the acute and chronic management of severe ectopic Cushing’s syndrome due to pNET. It also highlights the role of endoscopic ethanol ablation as a minimally invasive curative option for patients who are unfit for surgery.

## Introduction

Pancreatic neuroendocrine tumors (pNETs) constitute a rare subset of pancreatic neoplasms, comprising approximately 1–2% of all pancreatic malignancies (1). They are broadly categorized into non-functioning and functioning types, with non-functioning tumors constituting approximately 60–90% of all pNETs (2). The distinctive capacity of pNETs to synthesize and secrete various hormones and neuropeptides underlies the range of clinical syndromes associated with hormone hypersecretion (3). Ectopic adrenocorticotropic hormone (ACTH)-dependent Cushing’s syndrome (EAS) is a rare complication of pNETs that arises from the aberrant production of ACTH, leading to excessive glucocorticoid secretion (4).

EAS constitutes 10–20% of endogenous cases and most commonly originates from bronchial NET, small cell lung cancer (SCLC), thymic NET, pheochromocytoma and pancreatic NET (5).

Cushing’s syndrome (CS) is a clinical condition characterized by prolonged exposure to elevated cortisol levels. Chronic hypercortisolism severely impacts quality of life and increases mortality, as it is associated with a range of complications, including increased cardiovascular risk, metabolic disorders (such as impaired glucose tolerance, hypertension, dyslipidemia, and obesity), thromboembolic events, electrolytes disturbances, and neurological disorders (6, 7).

Early diagnosis and prompt initiation of therapy are of paramount importance. However, effective treatment remains a significant clinical challenge in a substantial subset of cases. The gold standard for the management of CS is surgical resection of the primary lesion responsible for excessive ACTH or cortisol production, which offers the potential for complete remission (8). In cases of life-threatening severe hypercortisolism (SH) as well as during the preoperative period, immediate pharmacological intervention aimed at normalizing cortisol levels is critical and serves as a bridging therapy (9).

Long-term follow-up revealed that achieving sustained biochemical control of hypercortisolism, particularly in its severe forms, remains difficult (10). The efficacy of hypercortisolism management in emergency cases can be enhanced through dual blockade of cortisol synthesis (11). A novel and promising therapeutic approach involves the use of etomidate, a reversible inhibitor of 11β-hydroxylase (CYP11B1), which also inhibits 17α-hydroxylase/17, 20-lyase and cholesterol side-chain cleavage enzyme, in combination with osilodrostat, a potent oral inhibitor of CYP11B1. This strategy offers an effective means of achieving biochemical control of hypercortisolism in adults with CS (12).

This article presents a rare case of a female patient who developed ectopic CS caused by an ACTH-producing pNETs. The patient was treated with etomidate and osilodrostat to normalize hypercortisolism, followed by endoscopic ethanol ablation of the focal pancreatic lesion, which led to partial tumor regression, resolution of EAS, and discontinuation of osilodrostat.

## Case report

In March 2022, a 74-year-old female patient was admitted to the Department of Endocrinology at the Pomeranian Medical University in Szczecin, Poland because of significant deterioration in her general condition and severe hypokalemia.

The patient’s medical history included atrial fibrillation, chronic heart failure, type two diabetes, ischemic stroke approximately 5 years earlier, hypothyroidism following thyroidectomy for nodular goiter in 1997 (levothyroxine substitution), prior hysterectomy for uterine fibroids, and a left adrenal mass (19 × 15 mm).

In November 2021, the patient was diagnosed with a neuroendocrine tumor (NET) of the uncinate process of the pancreas (21 × 29 × 31 mm), which was confirmed by fine-needle aspiration biopsy. Due to the limited biopsy material, the Ki-67 index could not be determined. Somatostatin receptor scintigraphy, performed in December 2021, revealed a single focus of increased receptor uptake corresponding to the pancreatic lesion. Given the patient’s significant multimorbidity and frailty, she did not qualify for surgical resection. In January 2022, treatment with lanreotide, a somatostatin analog, at a dose of 120 mg subcutaneously every 4 weeks, was initiated.

On admission to our department 2 months later, in March 2022, the patient presented with weakness, cognitive deterioration, memory and speech impairment, worsening choreiform movements of the upper limbs, marked muscle weakness, thin skin with extensive ecchymosis, and involuntary choreiform movements of the upper limbs and neck. According to the patient’s family, the symptoms had begun 1 week before admission.

Laboratory findings revealed: hypokalemia (K^+^ 3.18 mmol/L), severe hyperglycemia (glucose 450 mg/dL, HbA1c 10.3%), hypocalcemia (total calcium 2.19 mmol/L), elevated morning ACTH and cortisol levels (85.2 pg/mL and 81.1 µg/dL, respectively), disrupted circadian rhythm with midnight cortisol 70.6 µg/dL and urinary free cortisol (UFC) 1,011.6 (36,0–137,0 ug/24). Pituitary hormone levels indicated panhypopituitarism (**Table 1**).

**Table 1:** Laboratory tests from before the onset of the disease, from the initial days of hospitalization, and follow-up results approximately two years after the incident.

Laboratory parameter	December 2021 – February 2022	March 2022	September 2024	Standards
Leukocytes [10^9^/L]	6,69 (19.01.2022)	11,55*	4,53	3,98-10,04
Hemoglobin [g/dl]	14,6 (19.01.2022)	14,8*	12,9	11,2-15,7
Thrombocytes [tys/µl]	220 (19.01.2022)	171*	185	150-400
Sodium [mmol/l]	139 (01.02.2022)	141*	141	135-145
Potassium [mmol/l]	3,90 (01.02.2022)	3,18*	3,93	3,50-5,50
Total calcium [mmol/l]	–	2,19*	2,28	2,20-2,55
Ionized calcium [mmol/l]	–	1,09 (31.03.2022)	1,25	1,12-1,32
Glucose [mg/dl]	90,10 (17.12.2021)	340*	103	70-99
ACTH [pg/ml]	–	85,20	82,40	7,20-63,30
Cortisol [µg/dl]	–	81,100	9,900	4,82 – 19,5
UFC µg/24 h	–	1 011,60	48,35	in 2022: 36,00-137,00 in 2024: 11,50-102,00
TSH [µIU/ml]	2,460 (17.12.2021)	0,211*	2,490	0,270-4,200
FT4 [ng/dl]	1,59 (17.12.2021)	0,55*	1,26	0,93-1,70
FT3 [pg/ml]	2,26 (17.12.2021)	<0,391*	2,31	2,00-4,40
FSH [mIU/ml]	72,10 (17.12.2021)	3,95 (30.03.2022)	66,00	25,8-134,8 (after menopause)
LH [mIU/ml]	–	0,61 (30.03.2022)	26,60	7,7-58,5 (after menopause)
Estradiol [pg/ml]	–	76,00 (30.03.2022)	<5,00	5,0-54,7 (after menopause)
IGF-1 [ng/ml]	–	22,30 (28.03.2022)	71,60	53,70-161,00
Glycated hemoglobin	6,97	10,3	6,57	(4-6)

*Tests performed at the hospital emergency department on 25.03.2022. [ACTH, adrenocorticotropic hormone; UFC, urine free cortisol; TSH, thyrotropin; FT4, free thyroxine; FT3, free triiodothyronine; FSH, follicle-stimulating hormone; LH, luteinizing hormone; IGF-1, insulin-like growth factor-1]

Treatment included fluid and electrolyte repletion and insulin therapy. A continuous low-dose infusion of etomidate was initiated to rapidly reduce cortisol levels and manage the complications of hypercortisolism. This led to normalization of electrolyte parameters and a reduction in cortisol levels within the first week of hospitalization.

**Figure 1:**
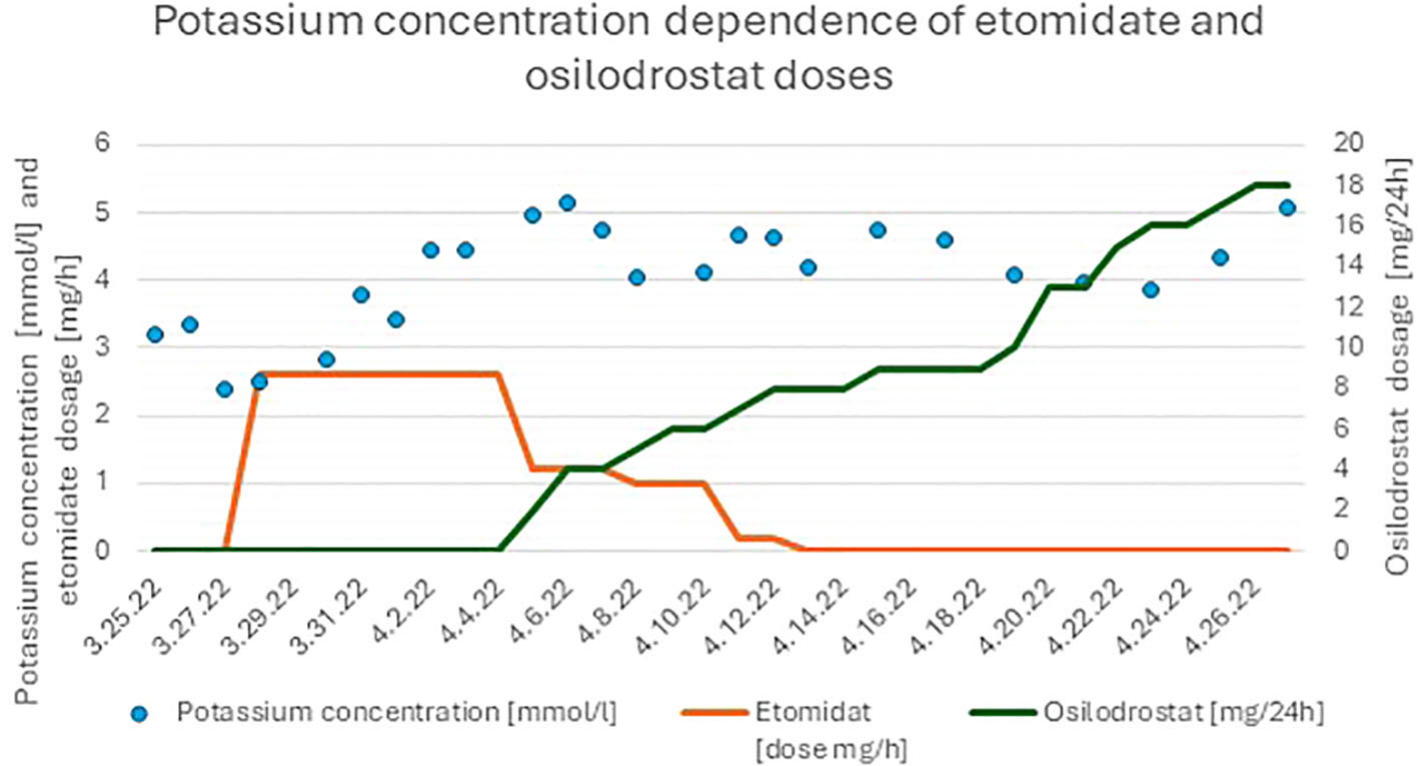
Changes in serum potassium concentrations during hospitalization and treatment with dual steroidogenesis blockade.

**Figure 2:**
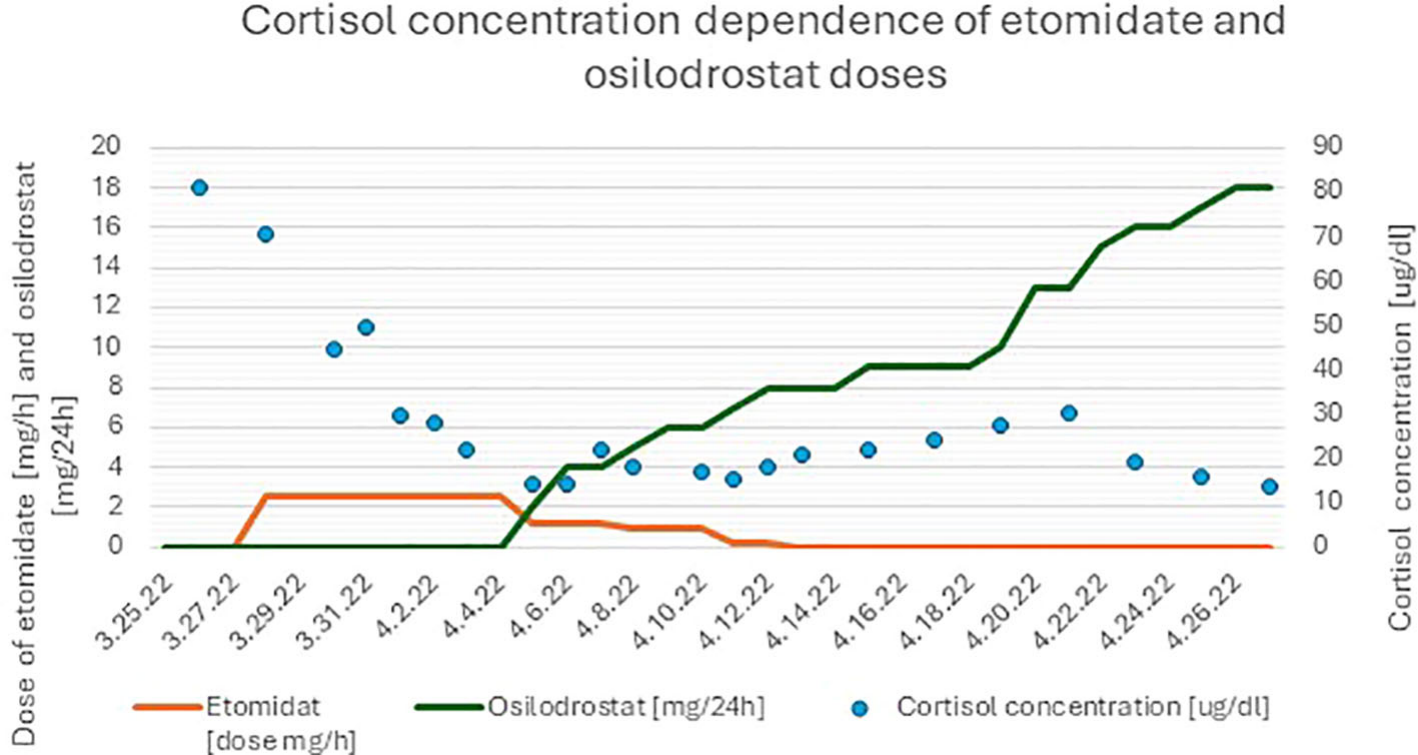
Changes in serum cortisol concentrations during hospitalization and treatment with dual steroidogenesis blockade.

Treatment with osilodrostat was initiated, as soon as available, on the 10th day of hospitalization. The dose was titrated to 18 mg/day, within 21 days (05.04.2022–26.04.2022). This oral steroidogenesis inhibitor successfully maintained cortisol suppression after tapering the etomidate administration on the 17th day of treatment (10). During the course of osilodrostat therapy, the patient continued to show significant clinical improvement; neurological symptoms resolved, cognitive function normalized, muscle strength increased, and she regained the ability to ambulate with a walker. By the 30th day of hospitalization, laboratory results confirmed ongoing hormonal control, with serum cortisol at 15.3µg/dL and potassium at 3.87 mmol/L (**Figures 1**, **2**).

Follow-up abdominal computed tomography (CT) on the 27th day of hospitalization revealed regression of the pancreatic tumor to 15 × 15 × 23 mm (previously 21 × 29 × 31 mm), with intense arterial-phase enhancement typical of pancreatic NET. The left adrenal lesion also decreased in size (from 19 × 15 mm to 14 × 14 mm), and both adrenal glands were irregularly thickened to 8–9 mm.

After discharge, lanreotide therapy (120 mg s.c. every 4 weeks) and osilodrostate 15 mg/day were continued. Gradual recovery of the pituitary gland was observed (**Table 1**). Both TSH and IGF-1 levels normalized within 1 month of hormonal control. FSH levels returned to normal, age-adjusted values 2 months after cortisol levels normalized.

**Figure 3:**
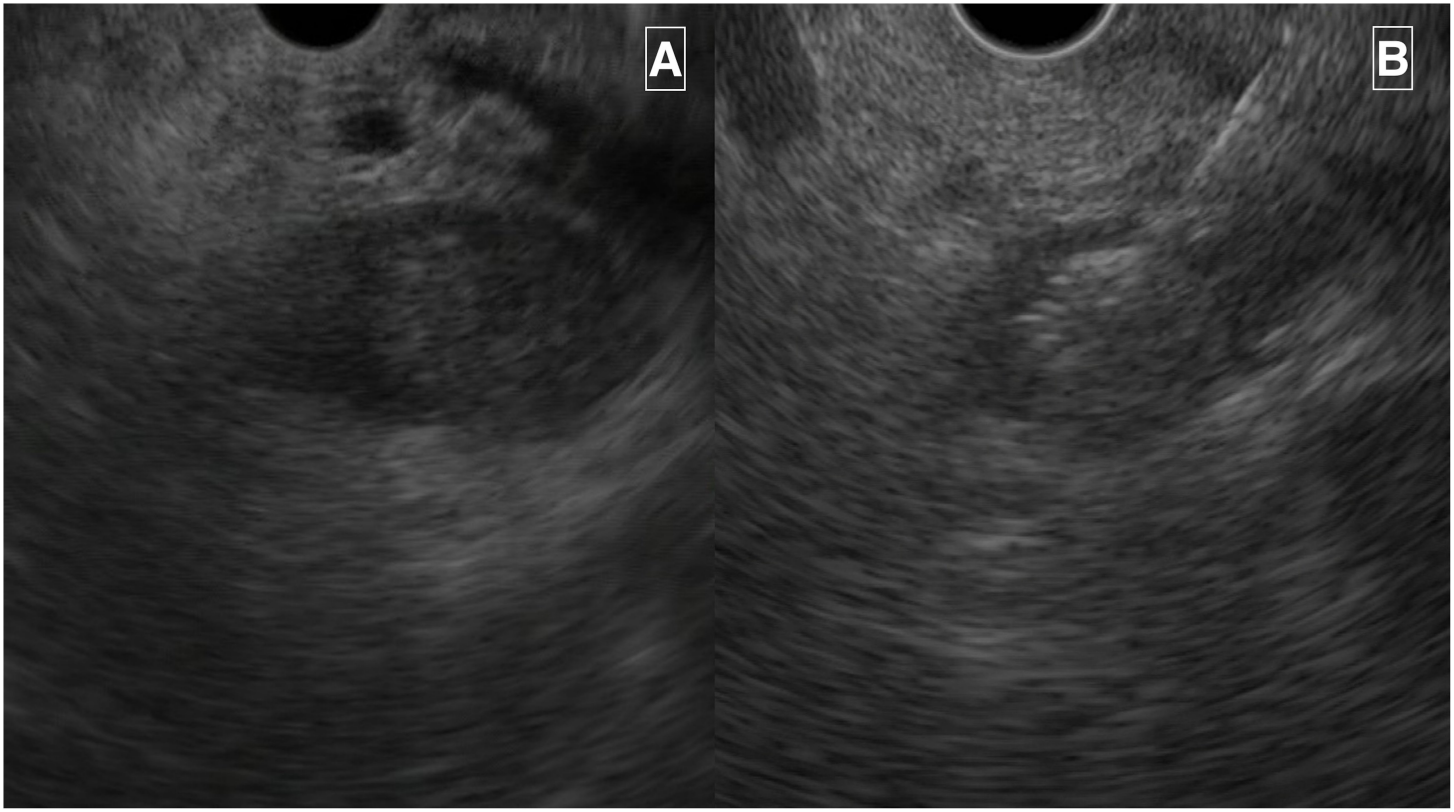
**(A)** Pancreatic neuroendocrine tumor visible on endoscopic ultrasound before ablation. **(B)** Pancreatic neuroendocrine tumor after administration of ethanol, with apparent change in its echogenicity.

After 19 months of osilodrostat treatment, endoscopic ultrasonography-guided ethanol ablation of the pancreatic tumor was performed. Transmural puncture and injection of 96% ethanol (2.5 mL) induced immediate echogenic changes in the lesions (**Figures 3A, B**). After ablation, osilodrostat was discontinued. ACTH levels gradually decreased over 5 months from 137 pg/mL before ablation to 75 pg/mL. Morning cortisol levels decreased from 11.1 µg/dL on osilodrostat treatment to 9.5 µg/dL without steroidogenesis inhibition. Subsequently, the patient experienced weight loss and fatigue. Tetracosatide stimulation test resulted positive (peak level 9.8 µg/dL) indicating hypocortisolism and the patient received hydrocortisone substitution.

Given the persistent positive somatostatin receptor imaging findings, lanreotide therapy was continued. The patient remained clinically stable and in good general condition. The hydrocortisone dose was gradually tapered and then discontinued.

## Discussion

The most commonly secreted hormones by functioning pNETs are gastrin and insulin (13). However, in rare cases, these tumors may produce ACTH, which can lead to the development of EAS (4). Ectopic CS accounts for 15% of all endogenous CS (14). This article presents a rare case of EAS managed successfully with multimodal treatment, including dual steroidogenesis inhibition and endoscopic ethanol ablation.

The typical symptoms of CS include depression, diurnal rhythm disturbances, muscle weakness, skin fragility, osteoporosis, central (truncal) obesity, a rounded “moon” face, hypertension and diabetes (15). The symptoms of EAS vary depending on the tumor type and the degree of cortisol elevation and typically cause a more gradual onset, while more aggressive histological types often lead to severe, rapidly progressing hypercortisolism and are more commonly associated with hyperpigmentation, weight loss, and mineralocorticoid effects (14, 15).

In a study by González Fernández et al., 12 patients with ectopic CS were described, and the most common clinical features included hypertension, diabetes mellitus, asthenia, and limb edema (14). Additionally, typical features of CS, such as proximal muscle weakness, capillary fragility, a Cushingoid phenotype, and weight gain, were frequently observed (14). Other less common symptoms include hyperpigmentation, virilization, menstrual disturbances in females, gynecomastia in males, psychiatric disorders, osteoporotic fractures, unusual infections, and nephrolithiasis (14). Laboratory tests often reveal hypokalemic metabolic alkalosis, resistance to treatment, leukocytosis, and elevated transaminase levels (14).

Our patient exhibited several clinical signs and laboratory abnormalities suggestive of EAS. The clinical picture was dominated by catabolic features, including myopathy, fragile skin, ecchymosis, hypokalemia, and neuropsychiatric symptoms (7, 16, 17). Nevertheless, the absence of metabolic syndrome and the rapid onset of symptoms made the diagnosis challenging. Other laboratory abnormalities, including leukocytosis and hypocalcemia supported the diagnosis of SH (18–20). Hormonal tests revealed high cortisol and ACTH levels, consistent with ACTH-dependent CS (21).

As ACTH and cortisol levels were not measured before the crisis, it is unclear how long the tumor was hormonally active. However, considering the initial findings from December 2021 (**Table 1**)—including controlled diabetes, normal leukocyte count, normokalemia, and no evidence of functional suppression of the pituitary, particularly within the gonadal axis—it is highly likely that ACTH secretion had an abrupt onset shortly prior to admission.

On admission, the patient presented with anterior pituitary insufficiency. Hypogonadism associated with hypercortisolism was recently investigated by Shekhar et al. (22). The authors demonstrated the suppression of LH pulse frequency in men with CS, which was reversible after achieving remission. In another study, menstrual abnormalities in women with CS were correlated with cortisol excess levels (23). The negative impact of hypercortisolism on the somatotropic and thyrotropic axes has been well described (24, 25). In our patient, pituitary function recovered within 8 weeks.

Androgen levels typically increase during the initial months of osilodrostat treatment; however, a decline has been observed in patients undergoing long-term exposure (26). Etomidate typically lowers estradiol and androgen levels due to the inhibition of 11β-hydroxylase. The unexpectedly high estradiol levels measured following initiation of etomidate and prior to implementation of osilodrostat most likely resulted from stimulation of the adrenal glands by ectopic ACTH and subsequent peripheral aromatization of adrenal androgens. Supporting this concept, the first testosterone measurement performed just 2 days after osilodrostat initiation revealed a highly elevated level (1,710 ng/mL). Both testosterone and estradiol levels substantially decreased during osilodrostat therapy.

Inferior petrosal sinus sampling (IPSS) is the gold standard for diagnosing the origin of ACTH-dependent CS (27). However, recent studies support the concept that, in cases of severe CS, the source of ACTH production may be determined using noninvasive techniques (28, 29). As shown by Lavoillotte et al., in patients with a UFC ten-fold above the upper limit of normal, a positive CT scan may be sufficient to confirm an ectopic source of ACTH (28). In the present case, the clinical context strongly indicated ectopic ACTH secretion, and pituitary MRI performed during follow-up did not reveal any focal lesions.

Although surgery remains the primary treatment for patients with CS, it is associated with an increased risk of complications and is not always feasible (11, 30). The pharmacological treatment of CS is complex and challenging, as no medication provides complete efficacy in managing hypercortisolism. Conventional therapy often proves ineffective in controlling life-threatening SH, with the use of individual drugs limited by the frequent side effects (10, 31).

In cases of severe CS, a promising strategy is a combination of osilodrostat, a selective 11β-hydroxylase inhibitor, with etomidate, a reversible non-selective inhibitor of the 11β-hydroxylase (the final step of cortisol biosynthesis), 17α-hydroxylase/17,20-lyase and cholesterol side-chain cleavage enzyme (10, 11, 32). Although etomidate is commonly used in the intensive care unit, it may also be used in acute settings where rapid cortisol control is required (33). In contrast, osilodrostat, owing to its selectivity, oral formulation, and long half-life, is particularly effective in the long-term control of cortisol levels in patients with hypercortisolism in the outpatient setting (10, 20, 34). Clinical studies have shown that osilodrostat is highly effective for normalizing UFC excretion and late-night salivary cortisol levels in patients with CS (35). However, the mean time to cortisol normalization in the randomized trial was 35 days. Therefore, the strategy of combining etomidate with long-acting osilodrostat as a bridging rescue therapy seems reasonable in patients with life-threatening SH (10, 32).

In our case, treatment with etomidate was initiated through a continuous low-dose infusion (5.5-7.5 mL/h), which resulted in a rapid decrease in serum cortisol levels and an improvement in electrolyte disturbances, particularly hypokalemia. As soon as available, osilodrostat was introduced following rapid dose adjustments with the intention of tapering the intravenous etomidate infusion. In summary, the combined use of etomidate and osilodrostat allowed both rapid and sustained control of ACTH-dependent hypercortisolism. The patient was ultimately discharged in good clinical condition and continued osilodrostat treatment at our outpatient clinic.

Surgical enucleation or resection remains the preferred treatment for pNETs. However, some patients are not eligible for surgery because of advanced age and significant comorbidities (36). Endoscopic ultrasound (EUS)-guided ethanol ablation was first performed in 2006 (37). It appears to be an alternative, local, and minimally invasive treatment option for solid pancreatic tumors, demonstrating high clinical efficacy, particularly for hormonally active pNETs (36, 38). This technique is suitable for selected patients and appears to be feasible, relatively safe, and effective, especially for alleviating symptoms by suppressing hormonal secretion (38). However, its use is limited by lesion size. The current knowledge of its efficacy in functional pNETs is based on case reports of insulinomas. The authors found no previous case reports of ACTH-producing pancreatic tumors treated using this technique.

Criteria for diagnosing remission in patients with pituitary ACTH-dependent CS have been well established, with morning serum cortisol values below 5 μg/dL (<138 nmol/L) or UFC below 28–56 nmol/d (<10–20 μg/d) within 7 days of selective tumor resection (39). However, there are currently no criteria for EAS remission. This is due to the rarity of the syndrome itself, lower rates of remission, and likely due to the more common use of steroidogenesis blockade before definitive treatment in this population. Considering existing case reports, the diagnosis of remission in EAS is usually made based on clinical improvement when confronted with low cortisol, ACTH, and UFC levels. Gamrat et al. described the remission of ectopic hypercortisolism in a patient with small lung cell cancer and a cortisol level 7.7 μg/L after discontinuation of osilodrostat (40). Jazdarehee et al. described remission of EAS in a patient with a medullary thyroid cancer after initiation of selpercatinib, with morning cortisol level of 6.23 μg/dL and requirement of steroid substitution (41). After tumor ethanol ablation, our patient developed subsequent and prolonged hypercortisolism despite withdrawal of osilodrostat, which is indirect proof of CS remission.

Remission of ectopic CS may be caused by the toxicity of steroidogenesis inhibition in the adrenal glands, cyclicity of ACTH production, or successive treatment of the secreting tumor. Sharma et al. presented four cases of ectopic CS remission as examples of different clinical scenarios (42). Although hypercortisolism in our patient may have had a sudden onset, cyclicity was less likely as the patient required osilodrostat for more than 1.5 years. Long-term dual steroidogenesis inhibition as an initial treatment, followed by prolonged exposure to osilodrostat, may have influenced the adrenal tissue. Varlamov et al. recently showed that osilodrostat treatment could induce adrenal shrinkage with or without adrenal insufficiency (43). The most recent abdominal CT scan (May 2025) demonstrated stable adrenal gland thickness (9 mm), but a reduction in the size of the focal lesion within the left adrenal gland, decreasing from 19 mm to 12 mm in diameter. Currently, the patient does not require steroid substitution, indicating that no permanent damage to the adrenal glands has occurred. In summary, as the patient experienced transient adrenal insufficiency after tumor ablation and withdrawal of osilodrostat, it is most likely that remission resulted directly from the ablative procedure.

## Conclusions

In patients with pNETs, clinical deterioration should raise the suspicion of hormonal activity. The treatment of severe CS requires both contemporary hormonal control and causal treatment, necessitating a multidisciplinary team. A combined dual steroidogenesis blockade may serve as a bridge therapy for patients with severe CS. Ethanol ablation may be a successful method for treating ectopic CS originating from localized pNET; however, further studies are required.

## Data Availability

The raw data supporting the conclusions of this article will be made available by the authors, without undue reservation.
